# The efficacy of genetic counseling for familial colorectal cancer: A meta‐analysis

**DOI:** 10.1002/jgc4.70046

**Published:** 2025-06-03

**Authors:** Andrada Ciucă, Tara Clancy, Sebastian Pintea, Ramona Moldovan

**Affiliations:** ^1^ Department of Psychology Babeş‐Bolyai University Cluj‐Napoca Romania; ^2^ Division of Evolution and Genomic Sciences, School of Biological Science University of Manchester Manchester UK; ^3^ Manchester Centre for Genomic Medicine, St Mary's Hospital, Manchester University Hospitals NHS Foundation Trust Manchester Academic Health Science Centre Manchester UK

**Keywords:** familial adenomatous polyposis, familial colorectal cancer, genetic counseling, Lynch syndrome, meta‐analysis

## Abstract

Genetic counseling has been proven to be effective for several conditions in relation to outcomes such as risk perception and knowledge. By statistically combining data from individual studies, a meta‐analysis can provide a precise estimate of an intervention's overall effect. The aim of this quantitative meta‐analysis was to assess the efficacy of genetic counseling for familial colorectal cancer and to explore characteristics that might influence the direction or magnitude of the relationship between the intervention and the outcome. We conducted an electronic search of literature published until March 2024. This identified 3150 articles, 30 of which met the inclusion criteria. Effect size parameters and sample sizes for all variables in each study were included. Results showed that genetic counseling has an overall statistically significant effect size of small magnitude (*d* = 0.234). Results indicate that genetic counseling is effective for affective (*d* = 0.162), cognitive (*d* = 0.298), and behavioral outcomes (*d* = 0.539); for individuals with a personal and/or family history; whether testing is diagnostic or predictive; and, with the exception of uncertain/uninformative results (4 studies), regardless of the testing results. Also, clinical/research teams that included a genetic counselor generated a significantly larger effect compared to teams without a genetic counselor. Our analysis showed that genetic counseling is effective for familial colorectal cancer. These results should encourage theoretical analyses and empirical studies exploring the process and rationale of genetic counseling from a more programmatic perspective.

**What is known about this topic**

Genetic counseling in cancer settings is known to improve knowledge related to cancer and genetics, and accuracy of risk perception, and affective outcomes such as anxiety.

**What this paper adds to the topic**

This is the first meta‐analysis done to assess the efficacy of genetic counseling for colorectal cancer. Results show an overall efficacy on affective, cognitive and behavioral outcomes, and moderation analysis showed that it is effective regardless of personal of family history, purpose of testing, and testing results.

## 1 INTRODUCTION

Advances in genetics research have enabled the identification of individuals affected by or at risk of a range of conditions (Collins et al., 2003). The main aims of genetic/genomic services include informing individuals of their hereditary risk, treatment, and/or management options, and providing them with support (Lerman et al., 2002; Wang et al., 2004). Genetic counseling is key in helping people understand and adapt to the medical, psychological, and familial implications of genetic contributions to disease (Resta et al., 2006).

Genes associated with an increased risk of colorectal cancer (CRC) have been identified over the last 3 decades (Aarnio et al., 1999; Lynch & de la Chapelle, 1999; Wells & Wise, 2017). Between 20% and 30% of cases of colorectal cancer are now known to be familial (Lichtenstein et al., 2000; Lorans et al., 2018; Valle et al., 2019), with a clear genetic factor identified in approximately 5% of cases (Jasperson et al., 2010; Lorans et al., 2018; Valle et al., 2019). There has been a marked increase in the need for genetic testing and genetic counseling for CRC, and this is expected to increase as more genetic and other risk factors are identified (Collins, 2001; Kinmonth et al., 1998; Klek et al., 2020; Wells & Wise, 2017).

Inherited conditions have direct implications not only for affected individuals but also for both close and extended other family members, and may therefore be experienced as being particularly stressful (Hadley et al., 2010; Lodder et al., 2001; Shiloh et al., 1990). The goals of genetic counseling for familial cancer include assisting individuals in understanding the inheritance pattern, increasing the accuracy of their perceived risk of developing cancer, providing information about genetic testing and management options so they can make informed choices regarding options available, and encouraging adherence to screening recommendations (Bowles Biesecker & Marteau, 1999; Braithwaite et al., 2006; Culver et al., 2024; Kessler et al., 2000).

Genetic counseling randomized controlled trials tend to target outcomes such as psychological wellbeing, knowledge, perceived risk, satisfaction, genetic testing or screening intentions, genetic testing uptake, decision making, medical management and health behavior, information sharing, and informed choice (Athens et al., 2017). Outcomes suggest a mixture of statistically significant and non‐significant findings but overall show a decrease in anxiety, depression, cancer‐related worry, decisional conflict, and perceived risk and an increase in knowledge (Culver et al., 2024; Madlensky et al., 2017). Numerous studies have shown that genetic counseling is beneficial in oncology settings for outcomes such as knowledge related to cancer (Braithwaite et al., 2006; Culver et al., 2024) and accuracy of risk perception (Braithwaite et al., 2006; Collins et al., 2008; Culver et al., 2024; Madlensky et al., 2017; Smerecnik et al., 2009). A meta‐analysis of the impact of genetic counseling in women at increased risk of hereditary breast cancer showed that genetic counseling leads to a significant reduction in general anxiety and improved accuracy of perceived risk (Meiser & Halliday, 2002). Similarly, a more recent meta‐analysis on BRCA‐related cancers showed that genetic counseling led to lower anxiety, depression, and worry, improved understanding of risk, and decreased intention for testing (Nelson et al., 2019). Available findings also suggest that genetic counseling can be useful in understanding the genetic implications of familial CRC not only when associated with genetic testing but also in the absence of available genetic testing or when test results are uninformative (Collins et al., 2000a, 2000b; Shiloh et al., 2008).

The main aim of our meta‐analysis was to explore whether and to what extent genetic counseling for familial CRC is effective and to analyze variables potentially influencing its effect. The objective was to present a balanced and impartial summary of the existing research that investigates the efficacy of genetic counseling for familial colorectal cancer through prospective and experimental study designs. A meta‐analysis achieves this by reviewing research findings through a quantitative lens, transforming the results from individual studies into an effect size, and then pooling and analyzing this information (Durlak, 2009). By assessing and combining the effect sizes of the different studies included, a meta‐analysis can provide a precise estimate of a medical or psychosocial intervention effect.

## 2 METHODS

### 2.1 Literature search

A literature search was conducted in the PubMed database to identify articles published until March 2024, without a defined starting point. The search included terms related to colorectal cancer, genetic counseling, and psychosocial interventions. The complete search syntax with all keywords included is presented in the Supplementary materials. Reference lists of articles from the full‐text assessment phase were manually searched to identify additional studies.

### 2.2 Inclusion/exclusion criteria

Inclusion criteria consisted of (1) research investigating the impact of genetic counseling for colorectal cancer, (2) published in English, (3) which explicitly used genetic counseling when describing the intervention, and (4) provided sufficient data to allow calculation of effect sizes (clinical trials and prospective studies). Studies were included regardless of the provider's background in genetic counseling to better capture areas where the genetic counseling profession is not fully established. Exclusion criteria consisted of (1) studies that included participants with various types of cancer (e.g., breast cancer) without providing a separate analysis for colorectal cancer patients; (2) studies with interventions provided to persons other than patients or family members (e.g., intervention on health professionals), and (3) studies analyzing the economic impact of genetic counseling. Two authors (AC and RM) independently assessed eligible studies.

### 2.3 Procedure and data analysis

For each study, the following variables were coded and extracted. Authors; year; number and mean age of participants; time points of the assessment (post‐intervention and follow‐up); type of outcome (affective, cognitive, behavioral, or quality of life); diagnosis (Lynch Syndrome, Familial Adenomatous Polyposis, familial CRC); cancer history (individuals who had a familial history of CRC or individuals who also had a personal diagnosis); genetic testing (individuals who had a genetic test and results were available, or who had a genetic test and results were not available and individuals who did not had a genetic test); purpose of testing (diagnostic test—if the participants were affected by cancer, predictive test—if the participants were not affected by cancer, both groups but results not given separately, and not tested); genetic status (Positive—carrier of a pathogenic/likely pathogenic variant; Negative—noncarrier of a pathogenic/likely pathogenic variant; Uncertain/Uninformative results—carrier of a variant of unknown significance or an uninformative result for an affected individual); and providing team background (with or without a genetic counselor included). Statistical data provided in studies were analyzed with Comprehensive Meta‐Analysis (CMA) software (version 2.2, 2011). The effect size for each study was automatically calculated by the software used based on the data available and introduced from each study. For pre‐post measures data (paired groups), if available, the effect size was calculated using the mean, standard deviation, and the sample size for each variable from the results section of the article; this was available for most of the studies included in this meta‐analysis. When means and standard deviations were not available, other modalities to calculate the effect size were used (i.e., the sample size and the *p*‐value, percentages of events and sample size, and number of events and sample size). Cohen's *d* coefficient was used to measure the effect size of the intervention, and its values are interpreted as small when *d* = 0.2, medium when *d* = 0.5, and large when *d* = 0.8. Data analysis was approached from 2 perspectives. First, the analysis looked at the overall effect of the intervention by comparing the pre‐intervention versus post‐intervention outcomes. Secondly, a moderation analysis was performed to identify potential variables that have a significant impact on the overall effect of genetic counseling.

For the purpose of the data analysis, the outcomes were grouped into 4 categories. In the affective outcomes category, the variables included were related to emotions and mood (e.g., anxiety, depression, fear of death or cancer, worry, emotional distress). In the cognitive outcomes category, the variables included were related to cognitive processes related to genetic counseling (e.g., knowledge, perceived risk, perceived self‐efficacy, commitment to screening, decisional conflict). In the behavior outcomes category, the variables included were related to screening behaviors such as colonoscopy adherence or endometrial screening. An additional quality of life category was set up as the concept encompasses a complex interaction between affective, cognitive, and behavioral outcomes. Variables included in this category were quality of life when measured directly and additionally other variables measured in a more general way such as mental health, impairment, and family communication aspects.

## 3 RESULTS

### 3.1 Literature search and characteristics of studies included

**FIGURE 1:**
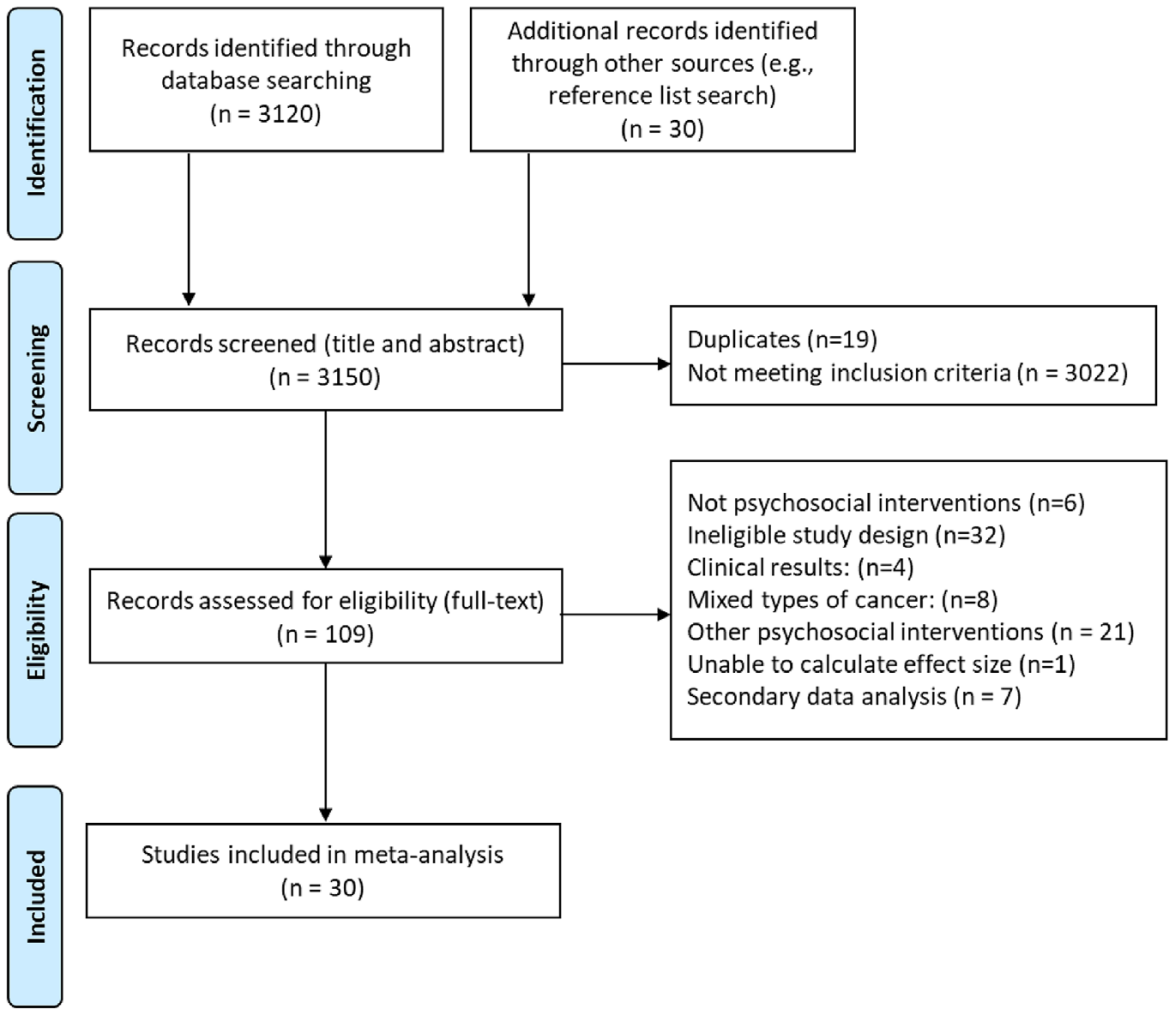
Flow diagram of the search.

The literature search identified a total of 3150 articles. 3120 articles were identified through database search and 30 through manual reference list search. After the screening of titles and abstracts, 19 articles were excluded as duplicates, and 3022 were excluded for not meeting the inclusion criteria (e.g., articles were focusing on CRC in general, with no connection to genetics/genetic counseling). After this, 109 articles were analyzed in full text format, and after applying the inclusion and exclusion criteria, 30 studies were included in the meta‐analysis. See Figure 1 for the flow diagram of the search procedure. Characteristics of eligible studies are presented in Table 1.

**TABLE 1:** Studies included in meta‐analysis.

No	Authors, publication year	Country	Study design	Diagnosis	Cancer history	Genetic testing	Purpose of testing	Report of mutation status	Outcome type	Provider	Mean age	% women	*N*
1	Aktan‐Collan et al. (2007)	Finland	Pre‐post	LS	Familial	Yes	P	NR	A, C QOL	nonGC	51.6	50	72
2	Aktan‐Collan et al. (2013)	Finland	Pre‐post	LS	Familial	Yes	P	P/N	A, C, QOL	nonGC	44.3	57.7	208
3	Arver et al. (2004)	Sweden	Pre‐post	LS	Familial	Yes	P	Mixed	A, QOL	nonGC	42.7	100	20
4	Burton‐Chase et al. (2013)	USA	Pre‐post	LS	Familial	Yes	P	P/N	C, B	GC	42	66	78
5	Claes et al. (2005)	Belgium	Pre‐post	LS	Familial	Yes	P	P/N	A	GC	39.25	40.27	36
6	Codori et al. (2005)	USA	Pre‐post	LS	Familial	Offered	P	NR	A, C	GC	43.8	58.4	101
7	Collins et al. (2000a)	Australia	Pre‐post	Mixed	Mixed	No	NT	NT	C	GC	46.7	67	126
8	Collins et al. (2000b)	Australia	Pre‐post	Mixed	Mixed	No	NT	NT	A	GC	47	67	127
9	Collins et al. (2005)	Australia	Pre‐post	LS	Familial	Yes	P	P/N/Mixed	B	NS	41.33	60.52	114
10	Collins et al. (2007)	Australia	Pre‐post	LS	Familial	Yes	P	P/N	A	NS	41	62	73
11	Dudok deWit et al. (1998)	Netherlands	Pre‐post	FAP	Familial	Yes	P	P/N	A	nonGC	28.6	61	23
12	Gritz et al. (1999)	USA	Pre‐post	LS	Mixed	Yes	D	P	A	GC	ns	ns	11
13	Gritz et al. (2005)	USA	Pre‐post	LS	Mixed	Yes	P & D	P/N/U	A, C, QOL	GC	ns	59.35	155
14	Hadley et al. (2004)	USA	Pre‐post	LS	Familial	Yes	P	P/N	B	GC	38.1	71	56
15	Hadley et al. (2008)	USA	Pre‐post	LS	Mixed	Yes	P	P/N	C, B	GC	37	100	65
16	[45] Hadley et al. (2011)	USA	Pre‐post	LS	Mixed	Yes	P & D	P	A, B	GC	41	52	129
17	Halbert et al. (2004)	USA	Pre‐post	LS	Familial	Yes	P	P	B	nonGC	49.3	68	71
18	Hasenbring et al. (2011)	Germany	Pre‐post	LS	Mixed	Offered	P & D	NR	A	nonGC	40.86	68.2	122
19	Johnson et al. (2002)	USA	Pre‐post	fCRC	Familial	Yes & No	P & NT	P/N/NT	B	NS	55	50.8	65
20	Keller et al. (2002)	Germany	Pre‐post	LS	Mixed	Offered	P & D	NR	A, C, QOL	nonGC	43.29	60	65
21	Keller et al. (2008)	Germany	Pre‐post	LS	Mixed	Offered	P & D	NR	A, C	nonGC	44	60.75	372
22	Loader et al. (2005)	USA	Pre‐post	LS	Mixed	Yes	D	P/U	A, C, B	GC	59.9	61.1	38
23	[52] Lynch et al. (1997)	USA	Pre‐post	LS	Familial	Yes	P	P	C	GC	ns	ns	20
24	Meiser et al. (2004)	Australia	Pre‐post	LS	Familial	Yes	P	P/N	A	NS	41.3	60.5	114
25	Murakami et al. (2004)	Japan	Pre‐post	LS	Mixed	Yes	P & D	P/N/U	A	nonGC	47	47.61	42
26	Pieterse et al. (2005)	Netherlands	Pre‐post	fCRC	Mixed	Offered	P & D	NR	A, C	nonGC	48.61	71.2	52
27	Rimes et al. (2006)	UK	Pre‐post	fCRC	Familial	No	NT	NT	A, C	nonGC	44.2	45	37
28	Shiloh et al. (2008)	USA	Pre‐post	LS	Mixed	Yes	P & D	P/N/U	A	GC	42.45	59	253
29	Voorwinden and Jaspers (2015)	Netherlands	Pre‐post	LS	Familial	Yes	P	P/N	A, C	NS	41.87	60.71	28
30	Wakefield et al. (2008)	Australia	RCT	LS	Mixed	Offered	P & D	NR	C, B, QOL	NS	50.5	57.79	109

*Note*: Diagnosis: LS, Lynch syndrome; FAP, familial adenomatous polyposis; fCRC, familial colorectal cancer; Mixed, LS + fCRC. Cancer history: Familial, Mixed, Personal + Familial. Purpose of testing: P, predictive; D, diagnostic; NT, not tested. Mutation status: P, positive; N, negative; U, uncertain or uninformative; Mixed, results not presented separately for positive and negative; NT, not tested; NR, tested; mutation status not reported. Outcome type: A, affective; C, cognitive; B, behaviors; QOL, quality of life; ns, not specified. Provider: GC, genetic counselor; nonGC, not genetic counselor; NS, not specified.

Of all studies, 12 were undertaken in the USA, 6 in Australia, 3 in Germany and the Netherlands, 2 in Finland, and 1 in Belgium, Japan, Sweden, and the UK. In terms of diagnosis in the 30 studies, 24 focused on Lynch syndrome, 3 on familial CRC (fCRC), 1 study on familial adenomatous polyposis (FAP), and 2 studies combined various diagnoses. Familial cancer history only was investigated in 16 studies, and individuals with a familial and a personal history of cancer were included in 14 studies. Genetic testing for either predictive (e.g., unaffected) or diagnostic (e.g., affected) purposes was provided in 28 studies, but the reporting of psychosocial outcomes depending on genetic testing results varied across the studies. Affective outcomes were measured in 20 studies, cognitive outcomes were measured in 15 studies, behavioral outcomes were measured in 9 studies, and quality of life outcomes were measured in 6 studies. Of the 30 studies, 13 clearly included genetic counselors in the providing team, 11 did not report a genetic counselor in the team, and in 6 studies data were not available; in the 11 studies where the background of the providers was known, 6 were medical geneticists, 1 was a psychologist, 1 was a medical oncologist or oncology nurse, and 3 studies had teams formed by medical geneticists, psycho‐oncologists, surgeons, and other professionals such as pathologists and molecular biologists. The mean age of the participants in the studies ranged between 28.6 and 59.9 years. The total number of participants included in the studies was 2782 individuals.

### 3.2 Overall results

**FIGURE 2:**
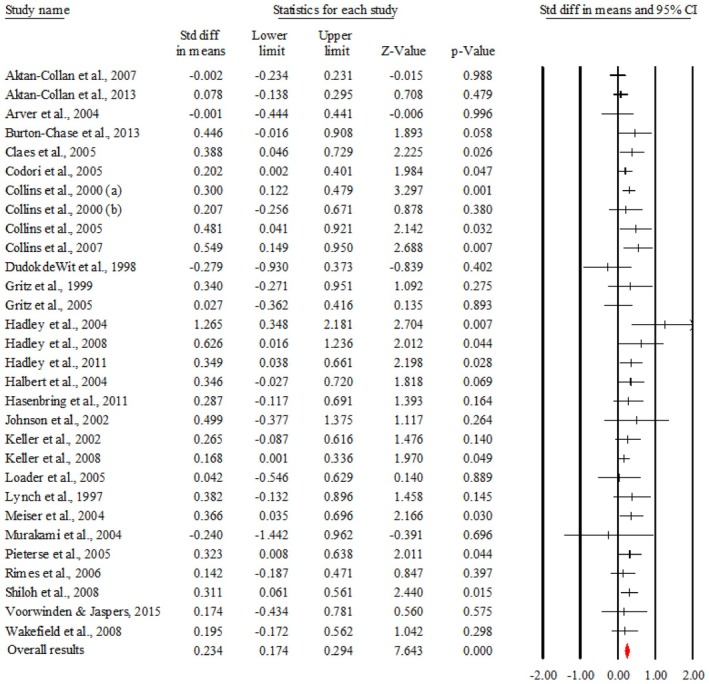
The overall results.

Genetic counseling has an overall statistically significant effect size, *d* = 0.234, *p* = 0.000 (95% CI [0.174, 0.294]), of small magnitude. Figure 2 shows the overall effect post‐intervention for each study included in the meta‐analysis.

### 3.3 Moderation analysis

A moderator is a characteristic that influences the direction or magnitude of the relation between the intervention and the outcome. Moderators clarify for whom or under what conditions an intervention works (Baron & Kenny, 1986). If genetic counseling outcomes vary as a function of different characteristics of the patient (e.g., diagnosis, genetic status, family history), the professionals delivering the session (e.g., genetic counselors or non‐genetic counselors) or the type of the outcome (e.g., affective, emotional, or behavioral), then these variables have a moderating impact on the outcome (Kazdin, 2009; Moldovan & Pintea, 2015). Table 2 presents the detailed results of the moderation analysis.

**TABLE 2:** Moderation analysis.

Moderator variable	Moderator categories	*N* (studies)	Cohen's *d*	Inf (95% CI)	Sup (95% CI)	*Z*	*p*	*Q*	df	*p*
Time point	Post	20	0.246	0.176	0.317	6.831	0.000	0.817	1	0.366
	Post and follow‐up	10	0.185	0.073	0.297	3.245	0.001			
Type of outcome	Affective	20	0.162	0.064	0.260	3.23	0.001	17.96	3	0.000
	Cognitive	15	0.298	0.188	0.409	5.28	0.000			
	Behaviors	9	0.539	0.352	0.726	5.64	0.000			
	QOL	6	0.056	−0.110	0.223	0.66	0.507			
Diagnosis	Lynch syndrome	24	0.229	0.161	0.297	6.63	0.000	2.83	3	0.418
	fCRC	3	0.253	0.033	0.473	2.25	0.024			
	Mixed	2	0.288	0.122	0.455	3.39	0.001			
	FAP	1	−0.279	−0.930	0.373	−0.83	0.402			
Cancer history	Familial	22	0.230	0.151	0.308	5.72	0.000	0.01	1	0.908
	Familial and personal	14	0.236	0.152	0.320	5.51	0.000			
Genetic testing	Yes	24	0.250	0.163	0.337	5.63	0.000	1.04	2	0.594
	No	4	0.104	−0.178	0.386	0.72	0.471			
	Offered	3	0.212	0.076	0.348	3.04	0.002			
Purpose of testing	Predictive	22	0.252	0.163	0.340	5.59	0.000	2.42	3	0.490
	Diagnostic	8	0.169	0.049	0.289	2.76	0.009			
	Not tested	4	0.104	−0.178	0.386	0.72	0.471			
	Both (P & D)	3	0.289	0.116	0.462	3.28	0.001			
Genetic status	Positive	19	0.232	0.107	0.357	3.64	0.000	11.50	4	0.021
	Negative	14	0.523	0.299	0.747	4.58	0.000			
	Not reported	9	0.171	0.080	0.261	3.69	0.000			
	Not tested	4	0.104	−0.178	0.386	0.72	0.471			
	Uninformative	4	0.060	−0.118	0.237	0.65	0.511			
Provider team with/without GC	With GC	13	0.304	0.215	0.393	6.69	0.000	5.25	1	0.022
	Without GC	11	0.159	0.072	0.245	3.58	0.001			

#### 3.3.1 Time points

There are no significant differences between studies with only post‐intervention assessment and studies that also included follow‐up assessments *Q*(1) = 0.817, *p* = 0.366. For studies measuring the effect at post‐intervention, the effect was small but significant *d* = 0.246, *p* = 0.000, and results are maintained at a statistically significant level when including follow‐up assessments *d* = 0.185, *p* = 0.001, which show that the outcomes of genetic counseling are maintained in time.

#### 3.3.2 Type of outcome

There is a significant difference between the categories of the outcome as moderator, *Q*(3) = 17.96, *p* = 0.000. The effect of genetic counseling upon affective (*d* = 0.162, *p* = 0.001) and cognitive outcomes (*d* = 0.298, *p* = 0.000) is small but significant, while the effect upon behaviors has a significant medium magnitude (*d* = 0.539, *p* = 0.000). Equally, the results indicate a non‐significant effect upon the quality of life (*d* = 0.056, *p* = 0.507).

#### 3.3.3 Diagnosis

There is a positive and significant effect for participants with Lynch syndrome (*d* = 0.229, *p* = 0.000), fCRC (*d* = 0.253, *p* = 0.024), and mixed diagnosis (*d* = 0.288, *p* = 0.001). Also, there is a negative low non‐significant effect size of genetic counseling for participants with a diagnosis of FAP (*d* = −0.279, *p* = 0.402).

#### 3.3.4 Cancer history

Both conditions of the cancer history moderator (i.e., familial only or familial and personal) obtained similar results, of small magnitude, with statistical significance. Genetic counseling had an impact of *d* = 0.230, *p* = 0.000 for individuals with a family history alone and an impact of *d* = 0.236, *p* = 0.000 for affected individuals with a family history.

#### 3.3.5 Genetic testing

There are no significant differences between genetic testing conditions. For individuals who were offered genetic testing, results show an effect size of small magnitude and statistically significant (*d* = 0.250, *p* = 0.000); results are similar for individuals who were offered genetic testing, but genetic testing results were not provided in the study (*d* = 0.212, *p* = 0.002). For individuals who were not offered genetic testing as part of the study, the results were non‐significant (*d* = 0.104, *p* = 0.471).

#### 3.3.6 Purpose of testing

There are no significant differences between the purpose of testing categories. For affected individuals who received diagnostic genetic testing, genetic counseling had an effect of *d* = 0.169, *p* = 0.009; for unaffected individuals who received predictive genetic testing, genetic counseling had an effect of *d* = 0.252, *p* = 0.000; and for studies where the results for diagnostic and predictive testing were reported combined, genetic counseling had an effect of *d* = 0.289, *p* = 0.001.

#### 3.3.7 Genetic status

There is a medium significant effect size of genetic counseling for participants with a negative result (*d* = 0.523, *p* = 0.000) and a small significant effect for patients with a positive result (*d* = 0.232, *p* = 0.000). For studies where genetic testing was offered but testing results were either not reported or were reported combined regardless of testing results, genetic counseling had an effect of *d* = 0.171, *p* = 0.000. Where genetic testing results were reported as uncertain or uninformative, genetic counseling had a non‐statistically significant effect size. Data also showed significant differences between these categories, *Q*(4) = 11.50, *p* = 0.021.

#### 3.3.8 Provider

There is a medium significant effect size of genetic counseling when provided with a genetic counselor in the team (*d* = 0.304, *p* = 0.000) and a small significant effect when provided without a genetic counselor in the team (*d* = 0.159, *p* = 0.001). Data also showed significant differences between these categories, *Q*(1) = 5.25, *p* = 0.022.

### 3.4 Methodological approach and publication bias analysis

We opted for two conservative methodological strategies to have a guarded and reliable analysis of the data: a random model of analysis and a thorough analysis of the publication bias. One aspect that needs to be considered when doing a meta‐analysis is the homogeneity or heterogeneity of the studies included. Our analysis showed that there is no significant heterogeneity of the results, *Q*(29) = 27.772, *p* = 0.530. Even with a non‐significant heterogeneity, a random model of analysis was used throughout in addition to moderation analysis due to the large methodological and theoretical diversity of the studies. Another threat to the validity of a meta‐analysis is publication bias (when studies with statistically significant or clinically favorable results are more likely to be published, and therefore analyzed, than studies with non‐significant or unfavorable results (Ahmed et al., 2012). We analyzed the publication bias for this meta‐analysis in two ways. First, we used the classic fail‐safe‐N, which suggests the number of studies with null effects that would bring the meta‐analysis effect to 0. Our meta‐analysis incorporates data from 30 studies, which yield a *z*‐value of 7.511 and corresponding 2‐tailed *p*‐value of 0.000. The fail‐safe N is 411. This means that we would need to locate and include 411 “null” studies for a combined 2‐tailed *p*‐value to exceed 0.050. In other words, this study would need to include 13.7 missing studies with null effects for every observed study, for the effect to be nullified. The second way to calculate publication bias is the Begg and Mazumdar Rank Correlation Test, which indicates if there is a relationship between the effect sizes and their standard error (which incorporates the size of studies). Our results show that there is no significant correlation, Tau = 0.094 (*Z* = 0.446, *p* = 0.464).

## 4 DISCUSSION

Our results show that genetic counseling is effective for familial CRC and has an overall statistically significant effect size of medium intensity. Results also show that genetic counseling for familial colorectal cancer is beneficial immediately after the session and at follow‐up, that is, the benefits gained during the genetic counseling session are maintained over time.

The results indicate that genetic counseling is effective for knowledge (understanding of causes, etiology) and psychological variables (anxiety, guilt, empowerment, self‐efficacy), for family members and patients, when delivered by genetic counselors and other professionals (effect sizes are statistically significant higher for genetic counselors). Data clearly show that genetic counseling is more effective in terms of improving knowledge than other psychological outcomes, such as emotional distress. This result may be explained by an effect on psychological outcomes being more difficult to achieve during an intervention with a small number of sessions (e.g., 1–3 sessions) and more vulnerable to change after the sessions (Nordmo et al., 2020).

Our results are in line with previous studies (Athens et al., 2017; Keller et al., 2008; Syngal et al., 2015) that have discussed or anticipated the potential role of genetic counseling for familial CRC. Our results are also comparable in terms of effect size with brief psychotherapy interventions (Nieuwsma et al., 2012). Moreover, previous meta‐analyses investigating the efficacy of genetic counseling showed comparable higher effect sizes for cognitive outcomes (e.g., knowledge) (Braithwaite et al., 2006; Culver et al., 2024) and lower or insignificant effect sizes for affective outcomes (e.g., anxiety and depression) (Braithwaite et al., 2006; Culver et al., 2024; Meiser & Halliday, 2002). This is not surprising as the content of genetic counseling sessions is more focused on less information‐giving (Meiser et al., 2008) and potentially less tailored to effectively address psychological constructs such as clinical anxiety or depression. Our results show limited effectiveness of genetic counseling for uncertain or uninformative test results based on 4 studies included in the analysis (Gritz et al., 2005; Loader et al., 2005; Murakami et al., 2004; Shiloh et al., 2008). The negative results of FAP cannot be extrapolated because they are based on the results of one study that included 23 participants and measured the impact of genetic counseling and testing with only a measure for emotional distress (i.e., Impact of Event Scale).

Our study highlights several important implications for clinical practice and future research. Firstly, our results help support evidence‐based practice and show that patients and their families are benefiting from empirically supported interventions. Secondly, only 1 study had a randomized control trial (RCT) design (Wakefield et al., 2008) where the primary focus of the trial was to investigate the efficacy of a decision aid added to genetic counseling. The other 29 had a pre‐post design. Future research based on well‐designed randomized clinical trials could allow both a more rigorous approximation of the efficacy of genetic counseling for familial colorectal cancer and the identification of ways to tailor the intervention to the specific needs of patients.

### 4.1 Limitations

The results of our meta‐analysis should be interpreted by taking into account several limitations of the included studies. Only 1 of the 30 studies included in the analysis had a control group and could rigorously attribute changes in scores to genetic counseling. We chose to include prospective designs because we were primarily interested in capturing a broad spectrum of the types of outcomes that have been studied. Future work would benefit from carefully considering the process of genetic counseling and the mechanism through which it is exerting its effect, as well as focusing on standardizing and harmonizing measures and outcomes included in the process. Another limitation to be considered is the timing of assessment in relation to the genetic counseling session, as between baseline and post‐test measures, participants could have been exposed to both genetic counseling and genetic testing, without having a clear assessment only after the genetic counseling session. Another aspect to be considered is that we could not evaluate the structure or quality of the genetic counseling sessions; the description of the interventions included in the analysis was variable, and there was not enough information to provide a comprehensive view of the aspects addressed during the genetic counseling session. Having said that, we did find clear indications that genetic counseling improves knowledge, alleviates emotional distress, and empowers patients and family members.

## 5 CONCLUSION

This is the first meta‐analysis investigating and showing the efficacy of genetic counseling for familial CRC. Our results may be particularly important given the early phase of the research in this area. Data showing the efficacy of genetic counseling provided in this meta‐analysis should encourage theoretical analyses and empirical studies exploring the process and rationale of genetic counseling from a more programmatic perspective.

## AUTHOR CONTRIBUTIONS

Authors confirm that they had full access to all the data in the study and take responsibility for the integrity of the data and the accuracy of the data analysis. All of the authors gave final approval of this version to be published and agreed to be accountable for all aspects of the work in ensuring that questions related to the accuracy or integrity of any part of the work are appropriately investigated and resolved.

## FUNDING INFORMATION

The work of Andrada Ciucă was supported through the Starting Research Grant financed by Babes‐Bolyai University (32968/23.06.2023), Romanian Minister of Education through the Agency for Credits and Scholarships, European Union's Horizon 2020 Research and Innovation Program (grant agreement 945151), and the LifeArc Pathfinder award (PATHFINDER004).

## CONFLICT OF INTEREST STATEMENT

Authors declare that they have no competing interests.

## ETHICS STATEMENT

Ethical approval was not required as the article analyzes data publicly presented in other published articles.

## Supporting information


Data S1

Data S2

## Data Availability

The datasets generated during and/or analyzed during the current study are available from the corresponding author upon reasonable request.
