# Supplementary material for: The efficacy of genetic counseling for familial colorectal cancer: A meta‐analysis
Source: J Genet Couns. 2025 Jun 3;34(3):e70046. doi: 10.1002/jgc4.70046 (PMC12131688; doi:10.1002/jgc4.70046)
Supplement: Supplementary file 2 — Data S2 [file JGC4-34-0-s001.docx]

**Supplementary Materials**

**Search syntax**

| Text box. 1 Search syntax |
| --- |
| ((((((((((((((((((colon cancer and psychological intervention)) OR (colon cancer and psychosocial intervention)) OR (colon cancer and psychotherapy)) OR (colon cancer and psychoeducational intervention)) OR (colon cancer and counseling)) OR (colon cancer and counselling)) OR (colorectal cancer and psychological intervention)) OR (colorectal cancer and psychosocial intervention)) OR (colorectal cancer and psychotherapy)) OR (colorectal cancer and psychoeducational intervention)) OR (colorectal cancer and counseling)) OR (colorectal cancer and counselling)) OR (bowel cancer and psychological intervention)) OR (bowel cancer and psychosocial intervention)) OR (bowel cancer and psychotherapy)) OR (bowel cancer and psychoeducational intervention)) OR (bowel cancer and counseling)) OR (bowel cancer and counselling) |
